# Successful biliary cannulation in complex anatomy: effectiveness of a novel rotatable sphincterotome in a case of billroth-I gastrectomy and periampullary diverticulum

**DOI:** 10.1055/a-2610-2638

**Published:** 2025-06-26

**Authors:** Shohei Kondo, Yasuhiro Kuraishi, Akira Nakamura

**Affiliations:** 1 Department of Gastroenterology, Shinshu University Hospital, Nagano, Japan

Biliary cannulation is a critical step in therapeutic endoscopic retrograde cholangiopancreatography (ERCP), but often poses challenges due to anatomical variations. In patients with Billroth-I gastrectomy, the duodenoscope tends to be positioned too closely to the papilla, which complicates cannulation with a standard catheter due to the difficulty in achieving an upward-facing view 1 . Moreover, in cases of periampullary diverticulum, the bile duct course frequently deviates, thereby complicating alignment with the duct axis 2 . A newly developed sphincterotome (ENGETSU, KANEKA Medix) offers enhanced rotational capability and adjustable blade angulation to enable precise adjustments in both vertical and horizontal planes ( Fig. 1 ). This functionality may be advantageous in overcoming the challenges from difficult anatomical orientations. We herein describe the successful implementation of this novel sphincterotome for biliary canulation in a patient with Billroth-I gastrectomy and periampullary diverticulum.

**Fig. 1:**
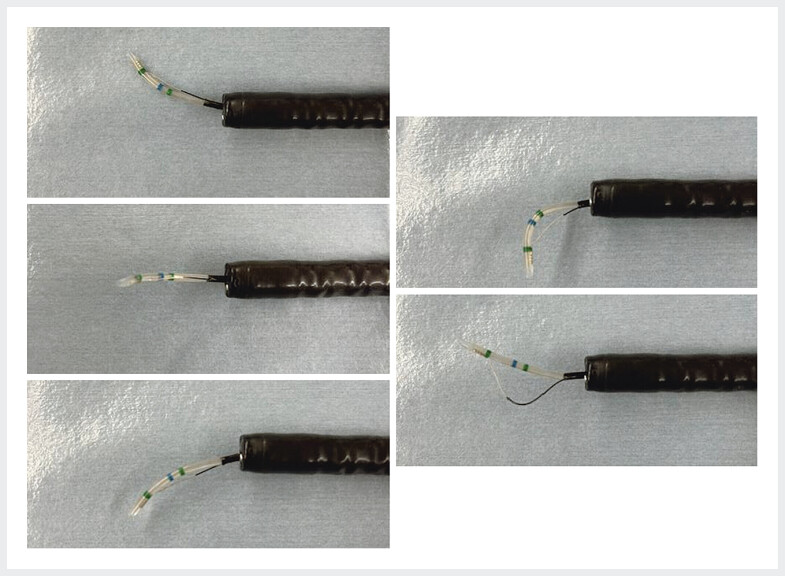
A newly developed sphincterotome (ENGETSU, KANEKA Medix) offers enhanced rotational capability and adjustable blade angulation to enable precise adjustments in both vertical and horizontal planes.

A 82-year-old man who had undergone Billroth-I gastrectomy presented with obstructive jaundice due to hilar cholangiocarcinoma and underwent ERCP for biliary drainage ( Fig. 2 , Video 1 ). The major papilla was located at the lower right edge of the diverticulum. Initial attempts at biliary cannulation using a standard ERCP catheter and a conventional sphincterotome were unsuccessful due to misalignment with the bile duct axis as being improperly oriented either downward or to the left. Employment of the two-devices-in-one-channel technique 3 was also ineffective. Biliary cannulation was then attempted using ENGETSU. By adjusting its rotation and angulation, the sphincterotome could successfully be aligned with the bile duct axis in vertical and horizontal planes to enable cannulation. Cholangiography revealed a hilar bile duct stricture. Following endoscopic sphincterotomy using ENGETSU, we performed trans-papillary biliary biopsy and drainage with a plastic stent.

**Fig. 2:**
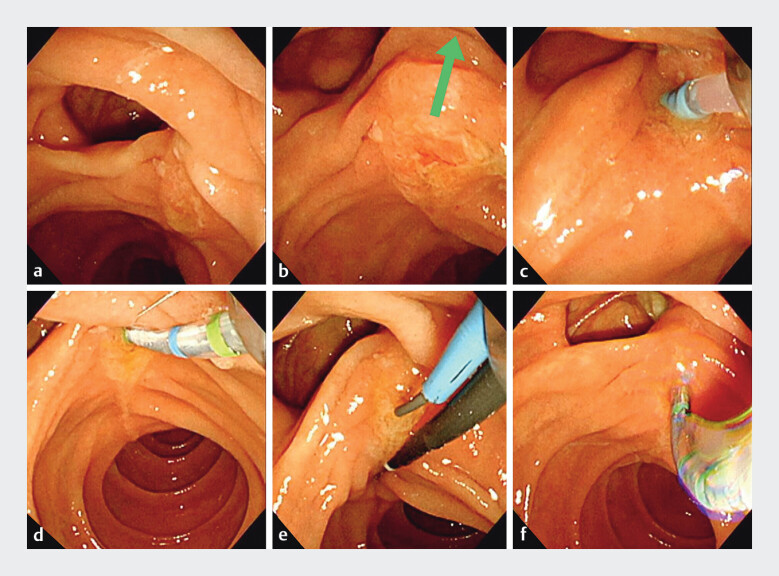
**a** The major papilla was located at the lower right edge of the diverticulum. **b** Arrow indicates the bile duct axis. **c** and **d** Initial attempts at biliary cannulation using a standard ERCP catheter and a conventional sphincterotome were unsuccessful due to misalignment with the bile duct axis by improper orientation either downward or to the left. **e** Selective biliary cannulation with the two-devices-in-one-channel technique was also ineffective. **f** By adjusting the rotation and angulation of the ENGETSU sphincterotome, close alignment with the bile duct axis was achieved in both vertical and horizontal planes, enabling successful cannulation. Abbreviation: ERCP, endoscopic retrograde cholangiopancreatography.

A novel rotatable sphincterotome enabled successful selective biliary cannulation in a challenging case of Billroth-I gastrectomy with periampullary diverticulum.Video 1

ENGETSU’s rotational and angulation capabilities provided precise control around the bile duct axis and facilitated biliary cannulation. This device may significantly improve the success rate of ERCP in cases of challenging anatomy.

Endoscopy_UCTN_Code_TTT_1AR_2AB

## Notes

Endoscopy E-Videos https://eref.thieme.de/e-videos
*E-Videos* is an open access online section of the journal *Endoscopy* , reporting on interesting cases and new techniques in gastroenterological endoscopy. All papers include a high-quality video and are published with a Creative Commons CC-BY license. Endoscopy E-Videos qualify for HINARI discounts and waivers and eligibility is automatically checked during the submission process. We grant 100% waivers to articles whose corresponding authors are based in Group A countries and 50% waivers to those who are based in Group B countries as classified by Research4Life (see: https://www.research4life.org/access/eligibility/ ). This section has its own submission website at https://mc.manuscriptcentral.com/e-videos .
